# Effectiveness and safety of pyrotinib‐based therapy in patients with HER2‐positive metastatic breast cancer: A real‐world retrospective study

**DOI:** 10.1002/cam4.4335

**Published:** 2021-10-21

**Authors:** Chao Li, Xiaoli Bian, Zhaoyun Liu, Xinzhao Wang, Xiang Song, Wei Zhao, Yansong Liu, Zhiyong Yu

**Affiliations:** ^1^ Cheeloo College of Medicine Shandong University Jinan China; ^2^ Breast Cancer Center Shandong Cancer Hospital and Institute Shandong First Medical University and Shandong Academy of Medical Sciences Jinan China; ^3^ Department of Breast Surgery Heze Municipal Hospital Heze China

**Keywords:** HER2‐positive, metastatic breast cancer, pyrotinib, real‐world study

## Abstract

The previous studies had demonstrated the promising effectiveness and acceptable safety of pyrotinib in patients with HER2‐positive metastatic breast cancer. We aimed to investigate the real‐world data of pyrotinib in complex clinical practice and complement the findings of clinical trials. Two hundred and eighteen patients were included for effectiveness analysis. A total of 62.0% had received two or more lines of systematic therapy, and 95.4% had been exposed to prior anti‐HER2 therapy, with 95.4% receiving trastuzumab, 5.0% receiving pertuzumab, and 40.8% receiving lapatinib. The median progression‐free survival (PFS) was 9.3 months and the objective response rate (ORR) was 44.0%. Patients treated with pyrotinib‐based therapy as first, second, or later line had a median PFS of 15.0, 10.3, and 6.8 months, respectively. Patients treated with pyrotinib and trastuzumab received significant benefit in terms of median PFS compared with pyrotinib alone (10.7 (9.1–12.3) vs. 8.8 (8.1–9.5), *p* = 0.016). Patients pretreated with lapatinib had a median PFS of 6.9 months. The median PFS time was 7.0 months in patients with brain metastasis. Multivariate Cox regression analyses showed that lines of pyrotinib‐based therapy (1 vs. 2 vs. ≥3), prior treatment with lapatinib, and combination treatments with trastuzumab proved to be independent predictors of PFS. Two hundred and forty‐eight patients were included in the safety analysis, and the results showed that the toxicity of pyrotinib was tolerable, with the most common grade 3/4 adverse event being diarrhea (19.8%). Pyrotinib‐based therapy demonstrated promising efficacy and tolerable toxicity in first‐, second‐, and later‐line treatments and in lapatinib‐treated patients. The combination of pyrotinib and trastuzumab showed advantages in PFS, even for patients resisting trastuzumab. Pyrotinib‐based therapy could be the preferred choice for brain metastasis patients, especially when combined with brain radiotherapy.

## Graphical abstract

Pyrotinib‐based therapy demonstrated promising efficacy and tolerable toxicity in first‐, second‐, and later‐line treatments and in lapatinib‐treated patients. Dual anti‐HER2 therapy with pyrotinib and trastuzumab showed advantages in PFS, even for patients resisting trastuzumab. Pyrotinib‐based therapy could be the preferred choice for brain metastasis patients, especially when combined with brain radiotherapy.

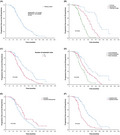

## 1 INTRODUCTION

Breast cancer with human epidermal growth factor receptor 2 (HER2) positivity occupied approximately 15%–20% of all breast cancers.^1^ It shows aggressive clinical behavior and has a poor prognosis. The development and widespread use of anti‐HER2‐targeted drugs have improved the outcomes of patients with HER2‐positive breast cancer.^2^ However, patients eventually develop resistance to these anti‐HER2‐targeted drugs and relapse. Therefore, it is necessary to develop novel anti‐HER2‐targeted drugs to overcome drug resistance.

Pyrotinib is an irreversible ErbB receptor tyrosine kinase inhibitor (TKI) that targets HER1, HER2, and HER4 and significantly improves the progression‐free survival (PFS) in HER2‐positive metastatic breast cancer (MBC).^3^, ^4^, ^5^ The PHOEBE study showed that pyrotinib plus capecitabine significantly improved the progression‐free survival (PFS; 12.5 vs. 6.8 months, *p* < 0.0001) and increased the objective response rate (ORR; 67.2% vs. 51.5%, *p* = 0.0091) and clinical benefit rate (CBR; 73.1% vs. 59.1%, *p* = 0.0155) compared with lapatinib plus capecitabine for HER2‐positive MBC pretreated with trastuzumab and chemotherapy.^5^ The PHENIX study included the HER2‐positive patients pretreated with taxanes, anthracyclines, and/or trastuzumab also confirmed the benefits of pyrotinib plus capecitabine with better PFS and ORR than capecitabine alone.^4^ Pyrotinib has been approved to be an alternative treatment option for patients with HER2‐positive MBC previously treated with trastuzumab and/or chemotherapy in China. Based on data from studies of pyrotinib, diarrhea and palmar‐plantar erythrodysesthesia (PPE) are the two most common complications occurring in patients receiving pyrotinib, leading to dose modification, treatment interruption, and even treatment discontinuation.

Although prior clinical trials have established the efficacy of pyrotinib in specific patients, they may fail to assess the complex conditions involved in routine clinical practice. Therefore, this real‐world study aims to fill a knowledge gap by investigating the effectiveness and safety of pyrotinib in complex real‐world clinical practice.

## 2 METHODS

### 2.1 Patients and treatments

This is a retrospective, single‐center study that enrolled patients with HER2‐positive MBC treated with pyrotinib at Shandong Cancer Hospital and Institute (Shandong, P.R. China) from October 2018 to October 2020. The eligibility criteria were as follows: (1) female patients with HER2‐positive MBC (HER2 positivity was histologically confirmed by immunohistochemistry category of 3+ or fluorescence in situ hybridization with HER2 gene amplification; if re‐biopsy of the metastatic site was infeasible, HER2 status was determined based on the latest primary tumor specimen); (2) not less than one measurable lesion based on Response Evaluation Criteria in Solid Tumors guidelines version 1.1 (RECIST 1.1); and (3) complete medical records. Patients were excluded from the study if they had previously enrolled in any pyrotinib‐related clinical trial settings or refused to provide written informed consent. Two hundred and forty‐eight patients enrolled the safety cohort for safety analysis. Patients who discontinued pyrotinib for severe adverse events or economic reasons and were lost to follow‐up were excluded from the efficacy analysis. Two hundred and eighteen patients were included in the efficacy cohort. The study was approved by the institutional review board, and all individual participants confirmed their informed consent.

Patients received pyrotinib in routine clinical practice, accompanied by chemotherapeutic drugs and/or anti‐HER2‐targeted agents. The initial dose of pyrotinib, dose modification, and treatment termination were determined by the physicians’ clinical decision according to clinical trial results, grade of adverse events (AEs), physical performance status, and willingness of the patient.

### 2.2 Assessments

Demographic and baseline data were collected, including age, histologic characteristics, line of treatment, metastasis site, previous treatment, medical history, and concomitant diseases.

The primary study endpoint was progression‐free survival (PFS), which was defined as the time from starting pyrotinib to disease progression or death from any cause. Central nervous system‐PFS (CNS‐PFS) was defined as the time from starting pyrotinib to intracranial progression or death. The objective response rate (ORR), overall survival (OS), and safety were the secondary endpoints. ORR was defined as the proportion of patients with complete response (CR) and partial response (PR). Tumor response was evaluated by RECIST 1.1 with physical examination and imageological examination. OS was defined as the time from starting pyrotinib until death, regardless of cause. Safety data were retrospectively collected based on medical records and laboratory test results, which are important identified risks for pyrotinib treatment in patients. AEs were graded using the National Cancer Institute Common Terminology Criteria for AE version 4.0. Trastuzumab resistance was defined as new recurrences diagnosed during or within 12 months after (neo)adjuvant trastuzumab or progression at first radiological reassessment or within 3 months after first‐line trastuzumab in the metastatic setting. Trastuzumab refractoriness is defined as progression after two or more lines of trastuzumab‐containing regimens that initially achieved disease response or stabilization at first radiological assessment. The disease‐free interval (DFI) was defined as the time from primary surgery to the diagnosis of metastasis.

### 2.3 Statistical analyses

Categorical variables were assessed by the Pearson's chi‐squared test or Fisher's exact test. Median DFS and OS were calculated using the Kaplan–Meier methodology, and univariate analyses were performed using the log‐rank test. A Cox regression model was performed using a stepwise selection of all factors studied as candidate predictors of PFS. Statistical analyses were performed using SPSS ver. 25.0 (SPSS Inc.). A *p* value of <0.05 was considered statistically significant.

## 3 RESULTS

### 3.1 Patient characteristics

A total of 267 patients with HER2‐positive advanced breast cancer were reviewed. Two hundred and eighteen patients were included in the efficacy cohort. The baseline characteristics are shown in Table 1. The median age at diagnosis was 51 years (range, 34–75 years) and 144 (66.1%) were premenopausal. The ECOG performance status was 0–1 in 203 (93.1%) patients at the time of therapy. The majority (191, 87.6%) of patients had IDC and the positivity of HR status was 52.8%. Thirty‐nine patients (17.9%) had de novo stage IV breast cancer.

**TABLE 1:** Baseline characteristics of patients

Characteristics	Patients, No (%) *N* = 218
Age, median (range years)	51 (34–75)
Menstrual status	
Pre‐menopausal	144 (66.1)
Post‐menopausal	74 (33.9)
ECOG performance status	
0–1	203 (93.1)
≥2	15 (6.9)
BMI	
<18.5	28 (12.8)
≥18.5, <25	99 (45.4)
≥25	91 (41.7)
Pathological type	
IDC	191 (87.6)
ILC	14 (6.4)
Other type	13 (6.0)
Grading	
1	12 (5.5)
2	70 (32.1)
3	136 (62.4)
HR status at metastatic setting	
Positive	115 (52.8)
Negative	103 (47.2)
Disease extent at diagnosis	
De novo IV stage	39 (17.9)
Metastatic	179 (82.1)
(neo) Adjuvant chemotherapy	
Yes	157 (72.0)
No	61 (28.0)
Adjuvant radiotherapy	
Yes	136 (62.4)
No	82 (37.6)
Adjuvant endocrine therapy	
Yes	87 (39.9)
No	131 (60.1)
Previous trastuzumab treatment	
Yes	
(neo) Adjuvant setting	82 (37.6)
Metastatic setting	186 (85.3)
No	10 (4.6)
Previous anti‐HER2 drugs	
Trastuzumab	208 (95.4)
Pertuzumab	11 (5.0)
Lapatinib	89 (40.8)
DFI (months)	
≥1 years	109 (50.0)
<1 years	70 (32.1)
De novo	39 (17.9)
Metastatic sites	
Local sites	107 (49.1)
Lymph node	152 (69.7)
Bone	109 (50.0)
Visceral	159 (72.9)
Brain	53 (24.3)
No. of metastatic sites	
1	29 (13.3)
2	50 (22.9)
≥3	139 (63.8)
Trastuzumab resistance status	
Yes	
Resistance	55 (25.2)
Refractoriness	148 (67.9)
No	15 (6.9)
Lines of pyrotinib in metastatic setting	
1	33 (15.1)
2	50 (22.9)
≥3	135 (62.0)

Abbreviations: DFI, disease‐free interval; ECOG, Eastern Cooperative Oncology Group; HER2, human epidermal growth factor receptor 2; HR, hormone receptor; IDC, invasive ductal carcinoma; ILC, invasive lobular carcinoma.

The median number of metastatic sites was 3.0, with 107 (49.1%) patients exhibiting local involvement, 152 (69.7%) exhibiting node metastasis, 109 (50.0%) exhibiting bone metastasis, 159 (72.9%) exhibiting visceral metastasis, and 53 (24.3%) exhibiting brain metastasis.

All patients except 10 (4.6%) had previously received anti‐HER2 therapy, with 208 (95.4%) patients receiving trastuzumab, 11 (5.0%) patients receiving pertuzumab, and 89 (40.8%) patients receiving lapatinib. The patients were exposed to trastuzumab in either (neo)adjuvant (82, 37.6%) or metastatic settings (186, 85.3%). Of the patients with trastuzumab in the (neo)adjuvant setting, 71 received trastuzumab over a 1‐year standard schedule, while 11 received less trastuzumab therapy due to primary resistance, intolerable toxicity, or other reasons. Trastuzumab resistance and trastuzumab refractoriness occurred in 25.2% and 67.9% of patients in the efficacy cohort, respectively.

Thirty‐three (15.1%) and fifty (22.9%) patients received pyrotinib‐based therapy as first‐line or second‐line systematic treatment, respectively. A total of 135 (62.0%) patients received two or more lines of prior treatment for MBC before pyrotinib, representing the heavily pretreated group.

### 3.2 Treatment administration

Treatment administration is shown in Table 2. Most patients (211, 96.8%) were exposed to pyrotinib in combination with chemotherapy as a starting treatment. The most common chemotherapy regimens were capecitabine (76, 34.9%), vinorelbine (56, 25.7%), Abraxane (58, 26.6%), and other regimens (21, 9.6%). Seven (3.2%) patients received pyrotinib alone.

**TABLE 2:** Treatment administration

Treatment	Patients, No (%) *N* = 218
Combined regimens with pyrotinib	
Capecitabine	76 (34.9)
Vinorelbine	56 (25.7)
Abraxane	58 (26.6)
Other	21 (9.6)
No	7 (3.2)
Target regimens	
Trastuzumab and pyrotinib	54 (24.8)
Pyrotinib alone	164 (75.2)
Pyrotinib dosage	
Starting dosage (mg/day)	
400	196 (89.9)
320	14 (6.4)
240	8 (3.7)
Dose reduction (mg/day)	
400→320	18 (8.3)
400→320→240	7 (3.2)
320→240	2 (1.0)
Dose escalation (mg/day)	
320→400	3 (1.4)
240→320→400	1 (0.5)
240→320	2 (1.0)
Interruption of treatment	31 (14.2)
Combined regimens modification	
Capecitabine dose reduction	6 (2.8)
Trastuzumab termination	1 (0.5)

According to the results of previous clinical trials, pyrotinib was initially prescribed at the standard dose of 400 mg/day in 196 patients (89.9%), 320 mg/day in 14 patients (6.4%), and 240 mg/day in 8 patients (3.7%). After the initial pyrotinib‐based therapy, patients received a modified dose based on the presence of AEs, physical health status, and the willingness of the patient. Twenty‐seven patients experienced a dose reduction in pyrotinib and 31 patients interrupted pyrotinib treatment. The main reasons were serious AEs, including diarrhea, vomiting, nausea, and anorexia. Six patients experienced dose escalation of pyrotinib from 240 or 320 mg/d to 400 mg/d or 320 mg/d.

In addition, six patients experienced dose reduction in capecitabine due to intolerant PPE. Of the 54 patients with dual anti‐HER2 therapy, 1 patient terminated trastuzumab after 8 cycles due to a left ventricular ejection fraction (LVEF) reduction of 10% relative to baseline.

### 3.3 Efficacy in overall patients

**FIGURE 1:**
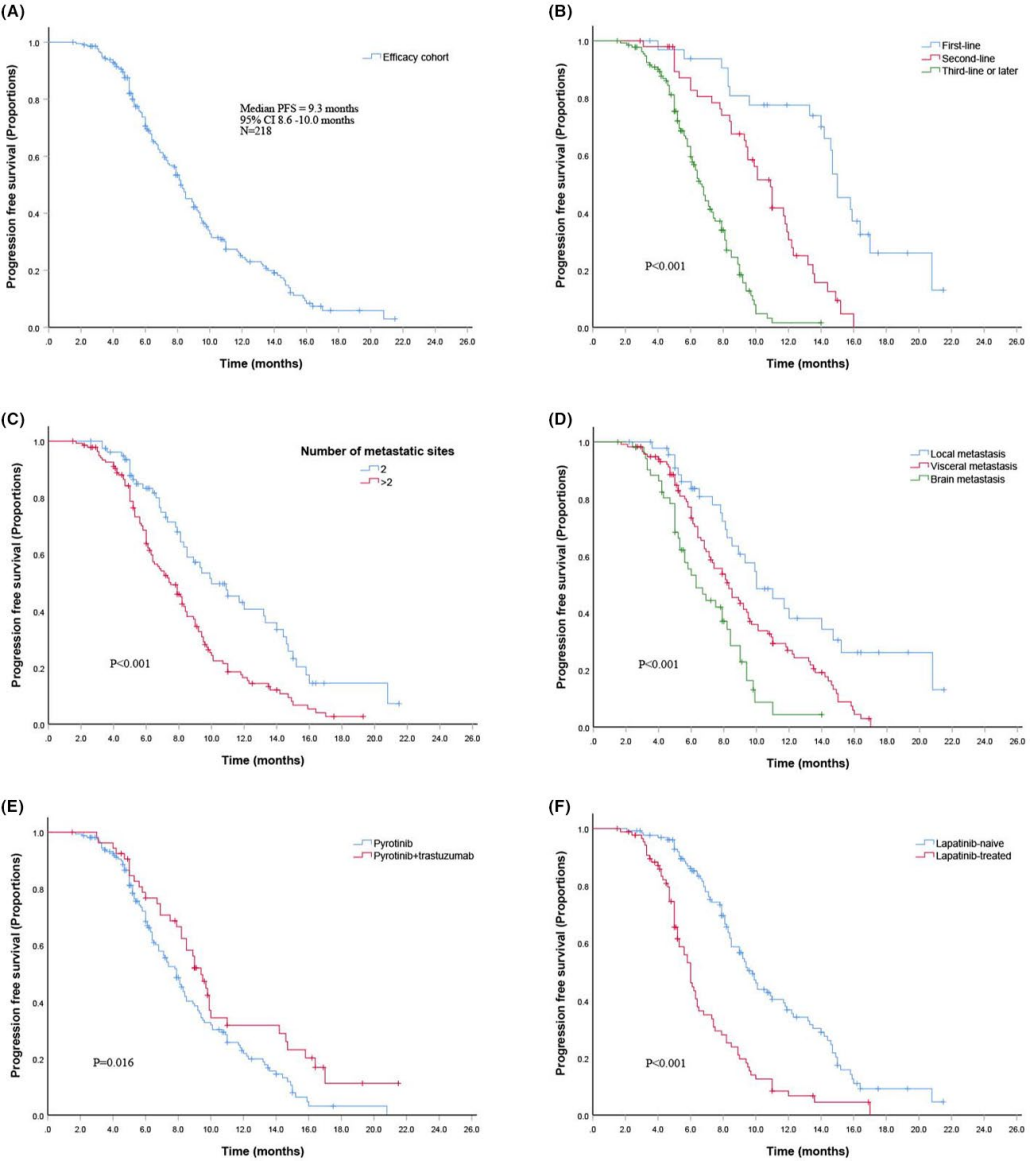
Kaplan–Meier curves of PFS for patients. (A) Overall cohort; (B) Patients stratified by treatment lines; (C) Patients with >2 or ≤2 metastatic sites; (D) Patients with different metastatic sites; (E) Patients treated with pyrotinib or pyrotinib+trastuzumab; and (F) Patients with lapatinib‐treated or lapatinib‐naïve

All patients were evaluable for PFS analysis. The median follow‐up time was 9.5 months (interquartile range, 5.0–10.0 months). The median PFS was 9.3 months (95% CI, 8.6–10.0 months) (Figure 1A). OS data were not achieved at the time of analysis.

Menstrual status, ECOG score, BMI, and hormone receptor status had no significant correlation with PFS in the log‐rank analysis (*p* = 0.638, *p* = 0.723, *p* = 0.751, and *p* = 0.968, respectively).

Patients who received pyrotinib‐based therapy as first, second, and later lines of metastatic treatment had a median PFS of 15.0 (13.2–16.8), 10.3 (9.3–11.3), and 6.8 (6.4–7.3) months, respectively (Figure 1B). Patients with one or two metastatic sites achieved a longer PFS time (11.4 (10.0–12.8) months) than patients with >2 metastatic sites (8.3 (7.6–9.0) months) (Figure 1C). Fifty‐three patients with brain metastases showed a median PFS of 7.0 (6.1–7.8) months. A total of 118 patients with visceral metastases, not including those who had brain metastases, and 47 patients with soft tissue and/or bone metastases only had median PFS of 9.1 (8.4–9.9) months and 12.3 (10.3–14.3) months, respectively (Figure 1D).

A total of 208 patients pretreated with trastuzumab showed a median PFS of 8.9 (8.3–9.6) months. A total of 203 patients received pyrotinib‐based therapy after exhibiting resistance to trastuzumab, resulting in a median PFS of 8.7 (8.0–9.3) months. The median PFS times were 9.7 months, 10.2 months, and 8.7 months in patients treated with pyrotinib combined with capecitabine, vinorelbine, and Abraxane, respectively. No significant difference in the median PFS time was observed among patients receiving these three different combined regimens (*p* = 0.274).

The ORR was 44.0%. Fourteen patients (6.4%) achieved a complete response, 82 (37.6%) achieved a partial response, 72 (33.0%) had stable disease, and 41 (18.8%) progressed. Nine (4.1%) patients lacked examination assessments. (Table 3).

**TABLE 3:** ORR for efficacy cohort and subgroup

Response	Efficacy cohort *N* = 218	Patients treated with pyrotinib and trastuzumab *n* = 54	Patients with brain metastasis *n* = 53
Best Response			
Complete response	14 (6.4)	2 (3.7)	1 (1.9)
Partial response	82 (37.6)	27 (50.0)	22 (41.5)
Stable disease	72 (33.0)	16 (29.6)	18 (34.0)
Progressive disease	41 (18.8)	8 (14.8)	12 (22.6)
Unknown	9 (4.1)	1 (1.9)	0
ORR	96 (44.0)	29 (53.7)	23 (43.4)

Univariate analysis indicated that lines of pyrotinib‐based therapy (1 vs. 2 vs. ≥3), pretreatment with lapatinib, number of metastatic sites (≤2 vs. ≥3), and dual anti‐HER2 therapy with pyrotinib and trastuzumab were significantly correlated with PFS in the log‐rank analysis. However, multivariable Cox regression analyses showed that lines of pyrotinib‐based therapy (1 vs. 2 vs. ≥3), prior exposure to lapatinib, and combination therapy with trastuzumab were independent predictors of PFS. (Table 4).

**TABLE 4:** Log‐rank and Cox multivariate analysis of factors associated with progression‐free survival

Characteristic	HR (95% CI)	Log‐rank analysis *p* value	HR (95% CI)	Cox multivariate analysis *p* value
Age group (<60 vs. ≥60)	0.7617 (0.5250–1.1051)	0.119	1.093 (0.548–2.181)	0.800
Menopausal status (Pre‐ vs. post‐)	0.925 (0.664–1.289)	0.638	1.039 (0.544–1.985)	0.908
Hormone receptor status (HR+ vs. HR‐)	0.994 (0.727–1.358)	0.968	0.983 (0.710–1.362)	0.918
Number of metastatic sites (≤2 vs. >2)	0.531 (0.387–0.727)	<0.001	1.030 (0.675–1.570)	0.892
Visceral metastasis (yes vs. no)	0.516 (0.370–0.719)	<0.001	1.476 (0.954–2.283)	0.080
Brain metastasis (yes vs. no)	0.498 (0.319–0.775)	<0.001	1.332 (0.871–2.037)	0.186
Lines of pyrotinib‐based therapy (1 vs. 2 vs. ≥3)		<0.001		< 0.001
1 vs. 2			0.112 (0.057–0.222)	
1 vs. ≥3			0.307 (0.191–0.494)	
Prior exposure to lapatinib (yes vs. no)	0.389 (0.271–0.559)	<0.001	2.390 (1.674–3.412)	< 0.001
Combination with trastuzumab (yes vs. no)	1.541 (1.106–2.148)	0.016	0.532 (0.350–0.809)	0.003

### 3.4 Efficacy of dual anti‐HER2 therapy (pyrotinib plus trastuzumab)

**FIGURE 2:**
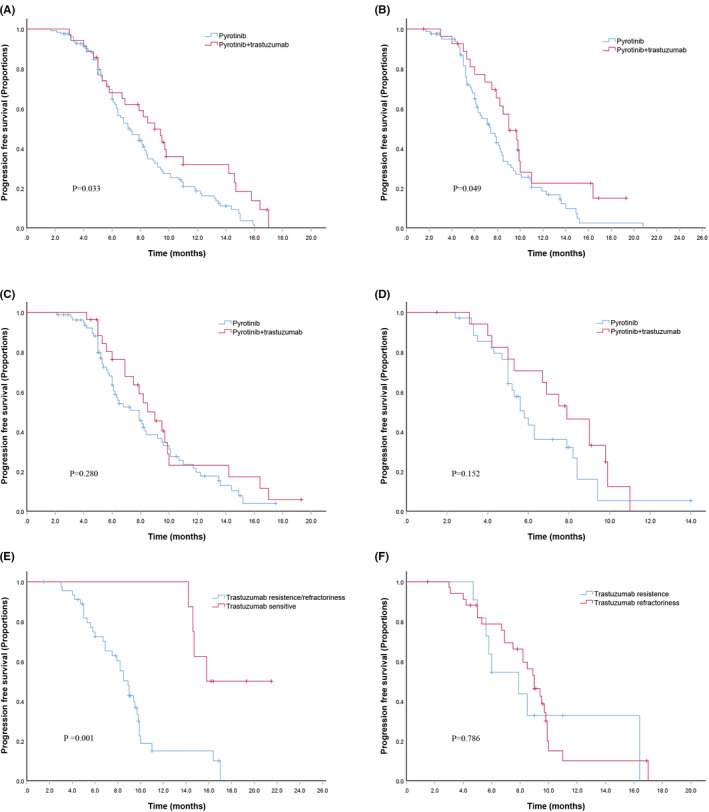
Kaplan–Meier curves of PFS for patients. (A) Visceral metastasis patients treated with pyrotinib or pyrotinib + trastuzumab; (B) Bone metastasis patients treated with pyrotinib or pyrotinib + trastuzumab; (C) Local metastasis patients treated with pyrotinib or pyrotinib + trastuzumab; (D) Brain metastasis patients treated with pyrotinib or pyrotinib + trastuzumab; (E) Patients treated with pyrotinib + trastuzumab for trastuzumab resistance/ refractoriness or sensitive; and (F) Patients treated with pyrotinib + trastuzumab for trastuzumab resistance or refractoriness

Fifty‐four (24.8%) patients received dual anti‐HER2 therapy (pyrotinib and trastuzumab) and chemotherapy. Dual anti‐HER2 therapy provided a significant benefit in terms of median PFS compared with pyrotinib alone (10.7 (9.1–12.3) vs. 8.8 (8.1–9.5), *p* = 0.016) (Figure 1E). Except for one patient who lacked an assessed examination, the remaining 53 patients achieved an ORR of 54.7%, with CR achieved in 2 patients and PR achieved in 27 patients. For patients with dual anti‐HER2 therapy, hormone receptor status and adjuvant anti‐HER2 therapy or not and number of metastatic sites had no significant correlation with PFS (*p* = 0.990, *p* = 0.580, and *p* = 0.081, respectively). Dual anti‐HER2 therapy had a better median PFS time than pyrotinib alone for patients with visceral metastasis (9.7 vs. 8.2 months, *p* = 0.033, Figure 2A) and bone metastasis (10.2 vs. 8.3 months, *p* = 0.049, Figure 2B), while not for patients with local metastasis (9.7 vs. 8.4 months, *p* = 0.280, Figure 2C) and brain metastasis (7.6 vs. 6.5 months, *p* = 0.152, Figure 2D). Median PFS time was significantly longer in patients without trastuzumab resistance and refractoriness than in those with trastuzumab resistance or refractoriness (18.2 vs. 9.0 months, *p* = 0.001) (Figure 2E). Patients with trastuzumab resistance had a similar PFS time to those with trastuzumab refractoriness (9.6 vs. 8.8 months, *p* = 0.786) (Figure 2F).

### 3.5 Efficacy in lapatinib‐pretreated patients

Eighty‐nine patients received lapatinib before pyrotinib‐based therapy. The median PFS time of patients with versus without lapatinib pretreatment was 6.9 (6.1–7.6) months versus 10.9 (10.0–11.9) months, respectively (*p* < 0.001) (Figure 1F). Eighty‐four patients were included in the ORR analysis, with an ORR of 46.4%. One patient achieved CR and 38 patients achieved PR. The baseline characteristics data showed that 74 (83.1%) patients were pretreated with more than two lines and 67 (75.3%) patients had ≥3 metastatic sites.

### 3.6 Efficacy in patients with brain metastasis

**FIGURE 3:**
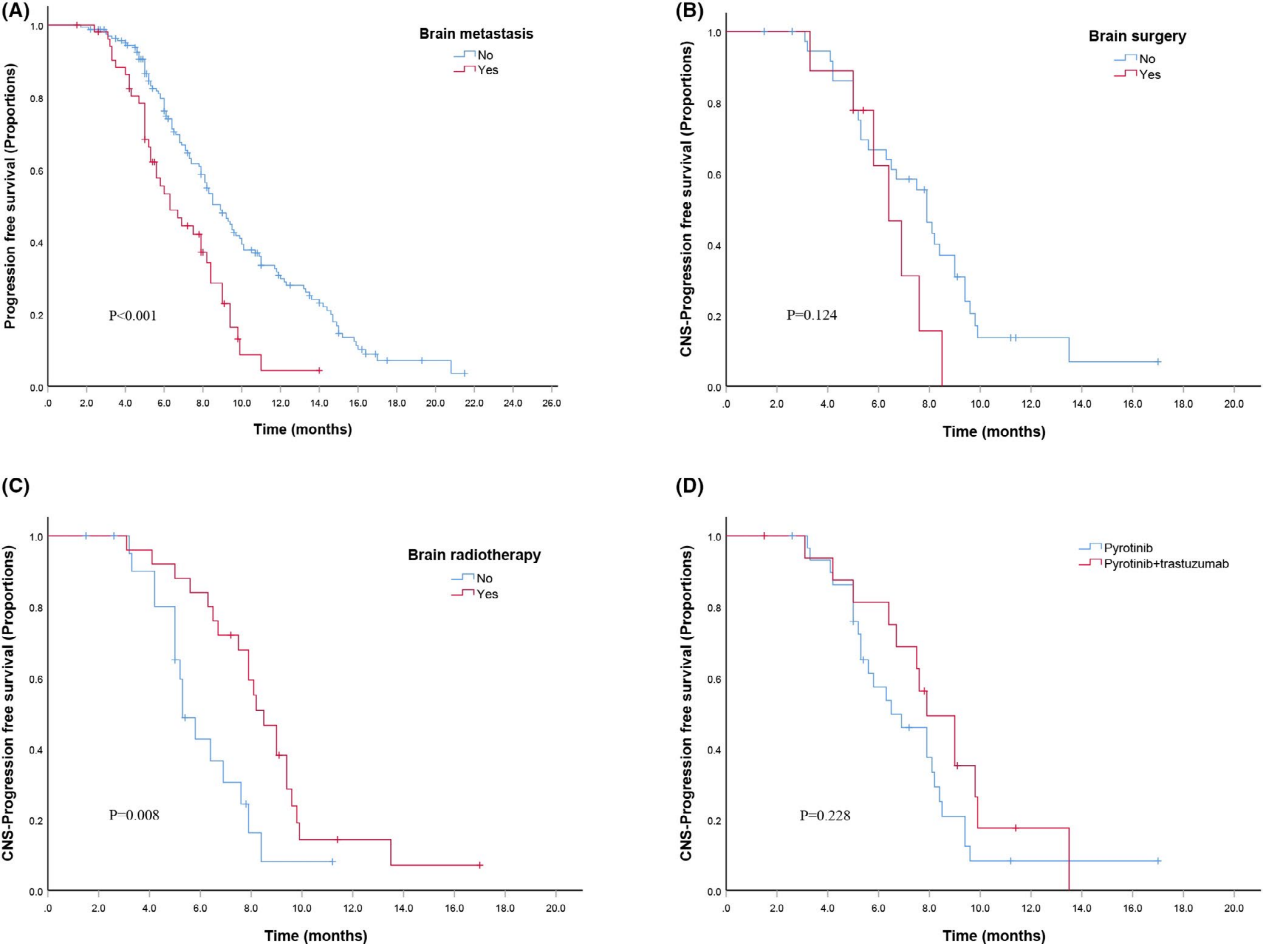
Kaplan–Meier curves of PFS for patients. (A) Patients with brain metastasis or not; (B) Brain metastasis patients treated with brain surgery or not; (C) Brain metastasis patients treated with brain radiotherapy or not; and (D) Brain metastasis patients treated with pyrotinib or pyrotinib + trastuzumab

Fifty‐three (24.3%) patients exhibited brain metastases. Patients with and without brain metastases had PFS times of 7.0 months and 10.0 months, respectively (*p* < 0.001) (Figure 3A). The ORR (regardless of intracranial and extracranial lesions) was 43.4%, with 1 patient achieving CR and 22 patients achieving PR. For intracranial lesions analysis, only 47 patients had measurable brain lesions. The ORR of intracranial lesions was 44.7% and CNS‐PFS time for patients was 7.9 months.

Of the 47 patients, 25 patients received brain radiotherapy (including whole brain and stereotactic radiotherapy) and 11 patients underwent brain surgery. The median CNS‐PFS showed no significant difference for patients underwent brain surgery or not (6.4 vs. 8.0 months, *p* = 0.124, Figure 3B). In patients who received pyrotinib‐based therapy and radiotherapy, the ORR was as high as 52%, while the brain radiotherapy‐naïve patients only got the ORR of 23% (5/22). The median CNS‐PFS time in the patients with versus without brain radiotherapy were 8.8 versus 6.2 months (*p* = 0.008), respectively, indicating a significant benefit from radiotherapy (Figure 3C). For patients with brain metastases, dual anti‐HER2 therapy with pyrotinib and trastuzumab had a similar CNS‐PFS time to pyrotinib alone (8.4 vs. 7.4 months, *p* = 0.228, Figure 3D).

### 3.7 Safety

The safety cohort comprised all patients who received at least one dose of pyrotinib and had available information, except those who refused to provide written informed consent. Two hundred and forty‐eight patients were enrolled in the safety analysis. As we retrospectively collected AE data from medical records and laboratory test results, the omission of AEs was unavoidable. The grades 3–4 AEs are presented in Table 5. The most common grade 3 and grade 4 AEs with standard pyrotinib were diarrhea (19.8%), PPE (6.9%), and neutropenia (4.8%). No treatment‐related deaths were reported. Overall, the safety of pyrotinib‐based therapy was controllable and tolerable.

**TABLE 5:** Adverse events (grade 3/4)

AE (grade 3/4)	Safety cohort (*N* = 248)	Pyrotinib and trastuzumab (*n* = 54)
Diarrhea	49 (19.8)	11 (20.4)
PPE	17 (6.9)	5 (9.3)
Neutropenia	12 (4.8)	4 (7.4)
Leukopenia	7 (2.8)	1 (1.9)
Thrombocytopenia	5 (2.0)	2 (3.7)
Anemia	9 (3.6)	1 (1.9)
Aminotransferase increased	10 (4.0)	2 (3.7)
Blood bilirubin increased	8 (3.2)	2 (3.7)
Rash	2 (0.8)	0
Vomiting	14 (5.6)	3 (5.6)
Fatigue	4 (1.6)	1 (1.9)
Dizziness	6 (2.4)	0
Mucositis oral	5 (2.0)	2 (3.7)
All	148 (59.7)	34 (63.0)

Abbreviation: PPE, palmar‐plantar erythrodysesthesia.

## 4 DISCUSSION

Dual anti‐HER2 therapy (trastuzumab and pertuzumab) and taxane has become the standard first‐line treatment for HER2‐positive MBC,^6^ and T‐DM1 was the preferred therapy for patients who exhibit progression after prior trastuzumab‐based treatment.^7^ However, pertuzumab and T‐DM1 were not approved for the metastatic setting in China, resulting in limitations in the ability to use these drugs in clinical practice. The preferred alternative is pyrotinib or lapatinib, especially for patients with resistance to trastuzumab. Based on the results of PHENIX and PHBOB studies, HER2‐positive MBC patients treated with pyrotinib plus capecitabine after trastuzumab and chemotherapy has been shown to improve the outcomes.^4^, ^5^ Major populations of HER2‐positive MBC patients previously treated with multiple anti‐HER2 therapies still respond to pyrotinib‐based treatment in clinical practice, and combined agents such as vinorelbine and Abraxane are incorporated into various chemotherapy regimens. Whether the efficacy and safety of clinical trials are applicable for later‐line pyrotinib‐based therapy remains questionable. Our study of a series of patients provides real‐world data to complement the results of previous clinical trials and a foundation to further explore pyrotinib treatment patterns.

In our study, the median PFS for all patients who received pyrotinib‐based treatment was 9.3 months, and the ORR was 44.0%, with a CR of 6.4% and PR of 37.6%, while the PFS time was 18.1 months and 12.5 months and the ORR was 67% and 78.5% for pyrotinib plus capecitabine, in PHENIX and PHBOBE studies, respectively. The disadvantages of our study may attribute to the enrollment of heavily pretreated patients who pretreated more than three lines of chemotherapy, but the two published studies only enrolled patients with up to two previous lines of chemotherapy for metastatic disease. Subgroup analysis suggested that patients treated with pyrotinib in first‐ and second‐line treatments achieved a longer PFS, indicating that prior line treatment affects the efficacy of pyrotinib‐based therapy.

Patients who received pyrotinib‐based first‐line treatment showed a median PFS of 15.0 months, which was shorter than that reported for trastuzumab and pertuzumab plus docetaxel in the CELEOPATRA study. Of the 33 patients receiving first‐line therapy, 13 patients were still undergoing treatment and 5 patients had brain metastasis. Compared with the CELEOPATRA study, more patients in our study had been pretreated with trastuzumab and had brain metastasis, suggesting that pyrotinib‐based therapy has great potential as a first‐line treatment for patients with trastuzumab resistance and brain metastases. Previous trials showed that patients receiving trastuzumab, pertuzumab plus capecitabine as a second‐line treatment reached a PFS of 11.1 months,^8^ which is even better than the PFS of 9.6 months in the EMILIA study.^7^ In our study, the PFS time was 10.3 months for the second‐line treatment, which was comparable to those reported from the aforementioned trial. For patients previously treated with at least two lines of anti‐HER2 therapy, pyrotinib‐based therapy achieved a median PFS of 6.8 months, which was slightly shorter than that of neratinib plus capecitabine (8.8 months) and comparable to lapatinib plus capecitabine (6.6 months) from the NALA study.^9^ Thus, pyrotinib‐based therapy also shows promise for patients with heavily treated HER2‐positive MBC.

Dual anti‐HER2 therapy with trastuzumab and TKIs has been proven to have enhanced antitumor activity in experimental and clinical studies, which is attributed to their different HER2 signaling targeting domains and synergistic drug interactions in neoadjuvant and metastatic settings. A phase III EGF104900 study explored the effectiveness of lapatinib plus trastuzumab in heavily treated HER2‐positive MBC and demonstrated a significant benefit to lapatinib monotherapy in terms of PFS (3 vs. 2 months, *p* = 0.008).^10^ The ALTERNATIVE study showed that patients receiving trastuzumab and lapatinib plus an aromatase inhibitor (AI) had better PFS than those receiving trastuzumab plus an AI (PFS: 11 vs. 5.7 months, *p* = 0.0064).^11^ Tucatinib, another small molecular TKI, showed promise in heavily pretreated patients with HER2‐positive MBC in the HER2CLIMB trial. The results showed that patients receiving dual anti‐HER2 therapy had a better median PFS and OS than trastuzumab monotherapy (7.8 vs. 5.6 months, *p* < 0.001; 21.9 vs. 17.4 months, *p* = 0.005), respectively.^12^ The results of our study also indicated advantages over pyrotinib monotherapy in terms of median PFS among patients receiving dual anti‐HER2 therapy (10.7 vs. 8.8 months, *p* = 0.016). Subgroup analysis showed that dual anti‐HER2 benefit from patients with visceral and bone metastases, not with local and brain metastases. For visceral and bone metastases, trastuzumab revealed its efficiency to extracellular HER‐2 receptor and got benefit with PFS. However, trastuzumab, a macromolecular monoclonal antibody, may be blocked by blood–brain barrier, which limited its effect to intracranial lesions. In addition, the advantage of dual anti‐HER2 therapy was maintained in both trastuzumab‐resistant patients and trastuzumab‐refractory patients, indicating its potential to reverse the resistance to trastuzumab. Moreover, trastuzumab and pyrotinib did not lead to an increased incidence of AEs compared with pyrotinib monotherapy.

Compared with lapatinib, pyrotinib can irreversibly bind to the intracellular tyrosine kinase domain and completely block the downstream signaling pathway. Because of the uniqueness of lapatinib as an anti‐HER2 TKI, before the approval of pyrotinib in China, some patients had already received lapatinib prior to anti‐HER2 treatment. ^13^ In our study, lapatinib‐naïve patients showed an advantage over lapatinib‐treated patients in terms of PFS time, which was attributed to their potential cross‐resistance to TKIs and sample differences in the treatment lines. The TBCRC022 study investigated the effectiveness and safety of neratinib plus capecitabine in HER2‐positive MBC patients, and the PFS was 3.1 and 5.5 months in lapatinib‐treated and lapatinib‐naïve cohorts, respectively.^14^ In our study, pyrotinib‐based therapy resulted in a median PFS of 6.9 months and an ORR of 43.8% in lapatinib‐treated patients, which was comparable to that from the TH3RESA study (6.2 months) and better than the 31% ORR reported in the T‐DM1 arm.^15^ Thus, pyrotinib‐based therapy is still an alternative for patients who previously received lapatinib‐based treatment.

In previous clinical trials of pyrotinib, the only choice for combination therapy was with capecitabine, which could not meet the needs of physicians in clinical practice. The LACOG 0801 study compared the effectiveness of lapatinib with capecitabine, vinorelbine, or gemcitabine in patients with HER2+ MBC, and the results showed that lapatinib plus capecitabine had a similar PFS to the other two regimens.^16^ Our results indicated that the PFS for patients received pyrotinib plus capecitabine was not significantly different from that with pyrotinib and vinorelbine, Abraxane or other chemotherapy, although the Abraxane arm had a lower median PFS than the other arms. Thus, we had more choices for regimens combining pyrotinib.

Overexpression of HER2 is associated with an increased incidence of brain metastases in breast cancer, which occurs in approximately 20%–50% of HER2‐positive breast cancers.^17^ Although treatment strategies range from local therapies to systemic anti‐HER2 therapies, the prognosis of patients with brain metastases remains poor. Anti‐HER2 monoclonal antibodies could not penetrate into brain in theory for their macromolecular properties,^18^ but trastuzumab and T‐DM1 still revealed promising efficacy in brain metastasis patients.^19^, ^20^ Compared with monoclonal antibodies, small molecular TKIs have become the preferred anti‐HER2 regimen for patients with brain metastases. In prior clinical trials, lapatinib or neratinib plus capecitabine achieved promising efficacy, with ORRs of 30% and 49% and median PFS times of 4.1 and 5.5 months in patients with brain metastasis from HER2‐positive MBC, respectively.^14^, ^21^ In the PHENIX study, pyrotinib plus capecitabine showed an PFS of 6.9 months for brain metastasis patients, which was better than that of lapatinib or neratinib numerically. In the HER2CLIMB trial, patients treated with tucatinib and trastuzumab plus capecitabine showed a median PFS of 7.6 months, and a 34% reduction in the risk of death was observed in heavily pretreated HER2‐positive MBC with brain metastasis.^12^ Compared with the PHENIX study and HER2CLIMB pyrotinib‐based therapy resulted in a median PFS of 7.0 months in our study, which is comparable to that of 6.9 months with pyrotinib plus capecitabine and less than that of 7.6 months with tucatinib and trastuzumab plus capecitabine. For intracranial lesions, the CNS‐PFS time was 7.9 months and the ORR was 44.7%, which is less than the CNS‐PFS of 9.9 months for tucatinib, trastuzumab, and capecitabine in the HER2CLIMB trial. Although the study enrolled patients with heavily pretreated HER2‐positive MBC in HER2CLIMB trial, dual anti‐HER2 therapy with tucatinib and trastuzumab may contribute to its favorable efficacy.

Previous studies found that the concentration of lapatinib was only 10%–20% of the concentration measured in extracranial secondaries. Hence, even though small molecule TKI drugs can theoretically reach intracranial lesions through the blood–brain barrier, the penetrability and distribution remain modest. Therefore, the pathophysiological changes following blood–brain barrier disruption may improve its permeability. Stereotactic or whole brain radiotherapy has been demonstrated to influence the efficacy of systemic anti‐HER2 therapy due to its damage to the blood–brain barrier, which allows for the regimen to penetrate.^22^ In a clinical study, stereotactic radiosurgery concurrent with lapatinib improved the local control rate of brain metastasis and reduced the risk of death without an increased rate of radiation necrosis.^23^ In our study, patients who only received pyrotinib‐based therapy without radiotherapy got the lower ORR of 23% (5/22), while the ORR of patients with pyrotinib‐based therapy and radiotherapy reached 52% (13/25). Meanwhile, patients treated with pyrotinib plus radiotherapy had a better median CNS‐PFS of 9.0 months than radiotherapy‐naïve patients (6.2 months). All these data suggest the great potential of pyrotinib‐based therapy in HER2‐positive MBC patients with brain metastasis, especially those who received concurrent or pretreated radiotherapy. However, brain surgery showed no benefit for the CNS‐PFS with pyrotinib for their limited influence to blood–brain barrier.

The incidence of AEs was similar to that reported in previous clinical trials of pyrotinib. All AEs were managed effectively by following the guidance and treatment advice regarding pyrotinib for patients. There was no difference between the incidence of grade ≥3 diarrhea in our study (19.8%) and in previous reports, which was consistent with the standard drug AE prevention literature. However, as dose reductions due to diarrhea accounted for half of the dose decreases reported in this work, continual monitoring for signs of diarrhea during pyrotinib treatment should be considered. The incidence of hand‐foot syndrome among patients in this work was lower than that reported in phase II and III clinical trials, which was attributed to the use of different combination drugs, such as vinorelbine and docetaxel, alone. The incidences of other AEs in our study were similar to those in previous clinical trials.

There were some limitations to our study. As a retrospective study, it is unavoidable that some clinical data were missed, resulting in information bias. The sample sizes of the brain metastasis group and dual anti‐HER2 group were small, and these conclusions should be further investigated by larger clinical trials. Moreover, the follow‐up time was rather short, and overall survival data were not mature enough to draw firm conclusions.

In conclusion, pyrotinib‐based therapy has demonstrated promising efficacy in HER2‐positive MBC with tolerable toxicity, regardless of whether it is administered as first‐, second‐, or later‐line treatment. Dual anti‐HER2 therapy with pyrotinib and trastuzumab achieved better efficacy than pyrotinib monotherapy, even in patients who were resistant to trastuzumab. In addition, the results revealed that pyrotinib‐based therapy had certain advantages for brain metastases, especially for patients who received brain radiotherapy concurrently and previously. Pyrotinib also confers benefits to lapatinib‐treated patients in terms of PFS time. In addition to capecitabine, pyrotinib also achieved similar efficacy in combination with other chemotherapeutic regimens, such as vinorelbine and Abraxane, in clinical trials. Besides, a number of prospective clinical trials on pyrotinib are undergoing, including combination with trastuzumab and chemotherapy in first‐line therapy of MBC and in neoadjuvant therapy. Part of these clinical trials have completed its enrollment and preliminary results had reported on some conferences. In the future, the final results may bring exciting results, so that pyrotinib can be applied in more treatment stages. More clinical trials are needed to further exploit the potential of pyrotinib‐based therapy.

## 5 ETHICS STATEMENT

The study involving human participants was reviewed and approved by the Ethics Committee and Institutional Review Boards in the Shandong Cancer Hospital and Institute. The participants provided their written informed consent in this study.

## CONFLICT OF INTEREST

The authors declare no conflict of interest.

## Data Availability

The datasets generated for this study are not publicly available due to hospital policy but are available upon reasonable request to the corresponding author.
